# Molecular characterization of Infectious Bursal Disease Virus isolated in Chile reveals several mutations in VP2 coding region and a reassortment in its genome

**DOI:** 10.1007/s11259-022-09956-x

**Published:** 2022-08-03

**Authors:** Miguel Guzmán, Leandro Cádiz, Alejandra Guerrero-Moncayo, Francisca Cáceres, Sonia Vidal, Lisette Lapierre, Leonardo Sáenz, Héctor Hidalgo

**Affiliations:** 1 Núcleo de Investigaciones Aplicadas en Ciencias Veterinarias y Agronómicas, NIAVA. Facultad de Medicina Veterinaria y Agronomía, Universidad de las Américas, Campus Maipu, Santiago, Chile; 2 Laboratory of Avian Pathology, Department of Animal Pathology, Faculty of Veterinary and Animal Sciences, Universidad de Chile, 8820808 Santiago, Chile; 3 Faculty of Chemical and Pharmaceutical Sciences, Faculty of Medicine, Advanced Center for Chronic Diseases Q5 (ACCDiS), University of Chile, Santiago, Chile; 4 Laboratory of Veterinary Vaccines, Department of Animal Biology, Faculty of Veterinary and Animal Science, Universidad de Chile, 8820808 Santiago, Chile; 5 Department of Animal Preventive Medicine, Faculty of Veterinary and Animal Sciences, Universidad de Chile, 8820808 Santiago, Chile

## Abstract

Infectious Bursal Disease (IBD) is a well-described disease in young chickens. It is caused by the Infectious Bursal Disease Virus (IBDV), which has a bi-segmented, double-strand RNA genome. The absence of a lipidic envelope makes IBDV highly resistant to environmental conditions. Consequently, it is widely reported around the world. Fourteen samples retrieved from chickens exhibiting apparent alterations of the bursa of Fabricius between 2017 and 2021 were included in the study. These samples were passaged into embryonated eggs and the presence of IBD was confirmed through RT-PCR. The PCR products were sequenced and analyzed to characterize the Chilean IBDV isolates for comparison with GenBank sequences, including vaccines sequences currently used in Chile.

Phylogenetic analysis classified the Chilean sequences as A1B1, except the sample 15002_CL_2021 which was classified as A2B1. On the other hand, all Chilean viruses were grouped as B1, based on viral segment B. Estimated evolutionary divergence between different genogroups supports these clustering. Moreover, samples 13936_CL_2017, 14038_CL_2017, 14083_CL_2017, 14145_CL_2018, 14431_CL_2019, and 14459_CL_2019 showed high similitude with the D78 and ViBursa CE vaccines (both currently used in Chile). Viruses 14010_CL_2018, 14040_CL_2017, 14514_CL_2019 and 14019_CL_2017 exhibited patterns that do not exactly fit either vaccine. Finally, viruses 15,041 N-_CL_2021, 15,041 N+_CL_2021, and 15004_CL_2021 showed even more differences regarding both vaccines.

This is the first study in Chile to analyze the genetic sequences of IBDV isolates. The different assessments conducted as part of the study suggest a close relationship with vaccines currently in use. Interestingly, one of the viruses exhibited a reassortment in its genome segments, which could confer new characteristics to the virus. However, new approaches would be required to establish the origin of the isolated viruses, as well as how the recombination is changing its virulence or morbidity.

## Introduction

Infectious Bursal Disease (IBD), also known as Gumboro disease, is a highly contagious infection affecting young chickens worldwide. Its etiological agent, the Infectious Bursal Disease Virus (IBDV), is a small viral particle with a diameter of 60 nm from the Birnaviridae family, Avibirnavirus genus (Dobos et al. 1979). As a non-enveloped virus, it is highly resistant to disinfectants and environmental conditions, making it very difficult to keep under control in commercial poultry flocks (Jayasundara et al. 2017).

IBDV has a double-stranded RNA (dsRNA) genome with two segments: A and B. Segment A, 3.2 kb in length, encodes for a polyprotein of 1,012 amino acids (aa), then cleaves into VP2, VP3 and VP4. VP2 is primarily responsible for producing neutralizing antibodies, as well as for facilitating the immune escape of IBDV. VP2 has also been proven to be a good predictor of phylodynamic relationships (Yilmaz et al. 2019). On the other hand, segment B encodes the viral RNA-dependent RNA polymerase (RdRp), VP1 (von Einem et al. 2004).

IBDV serotypes 1 and 2 have been described, but without effective cross-neutralization. Whereas serotype 2 has not been associated with IBD induction, serotype 1 has been classified into three different pathotypes. The first, ‘classical viruses’ are the most common in commercial poultry flocks. ‘Variant viruses’, which seem to be the product of antigenic drift from classical viruses, do not result in more mortalities but do cause immunosuppression issues in affected chickens. Finally, ‘very virulent viruses’ (vvIBDV) have caused high mortality rates in well-vaccinated flocks (Van Den Berg 2000). While vvIBDV was discovered in Europe, it has also subsequently been described in the Americas, Asia, and Africa (Hernández et al. 2006).

More recently, differences in virus genomes have been used to classify IBDV isolates from around the world into seven genogroups according to VP2 hypervariable regions of segment A (Michel and Jackwood 2017). The first three genotypes match the pathotypes mentioned above: classical, variant, and vvIBDV (Ali Khan et al. 2019). In 2021 Islam et al. (Islam et al. 2021) proposed a new classification, based on both viral genome segments. Thus, this new classification allows considering the incorporation of information regarding VP1, which contains the RNA-dependent RNA polymerase (RdRp) (von Einem et al. 2004), helping to understand changes in the virulence not related to immune evasion by changes in VP2. There are only a few reports of IBDV isolation in Chile (Jackwood and Sommer 1999; Noda et al. 2005), none of which underwent phylogenetic characterization. Therefore, this study aimed to describe and analyze IBDV strains isolated in Chile between 2017 and 2021 as well as their relationships to the vaccines currently used in the field.

## Materials and methods

### Samples

All the samples were obtained from chickens between two and six weeks old at Universidad de Chile’s Laboratory of Avian Pathology between 2017 and 2021. Seven IBDV isolate samples came from broilers and the other seven were from pullets. While no mortalities were associated with any of the cases, the bursa of Fabricius (BF) exhibited apparent alterations in the mucosa, while in those sampled in 2021 could be appreciated a higher inflammation damage. The birds came from different commercial poultry flocks in the Metropolitan, Valparaíso, and O’Higgins regions (Table 1).

### RNA extraction, RT-PCR, and sequencing

Using the PureLink™ Viral RNA/DNA Mini Kit (Invitrogen, USA), the RNA was extracted per manufacturer instructions from 500 µl of starting material obtained from a homogenized bursa of Fabricius. Between 676 bp and 1193 bp of the VP2 gene was amplified using the U3 (5’-TGT-AAA-ACG-ACG-GCC-AGT-GCA-TGC-GGT-ATG-TGA-GGC-TTGGTG-AC-3’) and L3 (5’-CAG-GAA-ACA-GCT-ATG-ACC-GAA-TTC-GAT-CCT-GTT-GCC-ACT-CTTTC-3’) primers, while VP1 gene of segment B was amplified using the + 290 (5’-TGT-AAA-ACG-ACG-GCC-AGT-GAA-TTC-AGA-TTC-TGC-AGC-CAC-GGT-CTC-T-3’) and − 861 (5’-CAG-GAA-ACA-GCT-ATG-ACC-CTG-CAG-TTG-ATG-ACT-TGA-GGT-TGA-TTT-TG-3’) primers, both of them recommended by the OIE (World Organization for Animal Health (OIE) 2019). Using SuperScript™ III Reverse Transcriptase (Invitrogen), cDNA was obtained per manufacturer guidelines from 2,5 µl of initial viral RNA. Using the Platinum™ SuperFi™ DNA Polymerase (Invitrogen), the PCR was conducted per manufacturer instructions with 5 µl of cDNA under the following parameters: 2 min at 94 °C, 40 cycles of 1 min at 94 °C, 30 s at 61 °C, 45 s at 72 °C; and, finally, an additional extension step at 72 °C for 10 min. The PCR products were visualized in an agarose gel and purified per manufacturer instructions with a PureLink™ Quick Gel Extraction Kit, then sequenced by Macrogen Inc. (Seoul, Korea).

**Table 1:** IBDV samples from the different chicken flocks utilized in this study

*ID*	*Isolation year*	*Type of bird*	*Diagnosis* */lesions*	*Origin*	*GenBank accession no.* VP2	*GenBank accession no. VP1*
14038_CL	2017	Broiler	Mild BF inflammatory lesion	Valparaíso	MW367227	OL744092
14040_CL	2017	Broiler	Mild BF inflammatory lesion	O´Higgins	MW367231	OL744093
13936_CL	2017	Broiler	Mild BF inflammatory lesion	Valparaíso	MW367233	OL744094
14083_CL	2017	Broiler	Mild BF inflammatory lesion	Metropolitan	MW367235	OL744095
14019_CL	2017	Pullet	Mild BF inflammatory lesion	Valparaíso	MW367236	OL744096
14145_CL	2018	Broiler	Mild BF inflammatory lesion	O’Higgins	MW367234	OL744097
14010_CL	2018	Broiler	Mild BF inflammatory lesion	O’Higgins	MW367230	OL744098
14514_CL	2019	Pullet	Mild BF inflammatory lesion	Valparaíso	MW367232	OL744099
14431_CL	2019	Broiler	Mild BF inflammatory lesion	Valparaíso	MW367228	OL744100
14459_CL	2019	Pullets	Mild BF inflammatory lesion	Valparaíso	MW367229	OL744101
15002_CL	2021	Pullets	High BF inflammatory lesion	Valparaíso	OL744106	OL744102
15,041 N-_CL	2021	Pullets	High BF inflammatory lesion	Valparaíso	OL744107	OL744103
15,041 A+_CL	2021	Pullets	High BF inflammatory lesion	Valparaíso	OL744108	OL744104
15004_CL	2021	Pullets	High BF inflammatory lesion	Valparaíso	OL744109	OL744105

### Dataset

The sequences obtained from each sample were assembled using Bioedit v 7.2.5 (Ahmed et al. 2013). They were all submitted to GenBank; accession numbers are listed in Table 1. The datasets were constructed using representative sequences from the genogroups previously described for segments A and B by Islam et al. (Islam et al. 2021).

### Analysis of hypervariable regions

The nucleotide sequences were translated into amino acid sequences using Bioedit v.7.2.5 software (Ahmed et al. 2013). The representative Chilean VP2 sequences were compared with vaccines currently used in Chile. The first sequence was the Vibursa CE vaccine (accession number EU201689), which is commercialized by Elanco ™ (https://www.elanco.us/); the second one was the D78 vaccine (accession number AJ586963), commercialized by MSD Animal Health © (https://www.msd-animal-health.com/). Both of them are freeze-dried lives vaccines containing a classic strain of Infectious Bursal Disease virus, their administration is through drinking water, spray, or eye drop/nasal instillation. Moreover, two sequences isolated from America were included in the comparison.

### Phylogenetic analysis

The datasets were aligned using Mafft v.7.2 software (Katoh and Standley 2013). The nucleotide substitution model was then chosen using JModelTest v.2.1.7 software (Darriba et al. 2012) and recombinant sequences were eliminated using RDP v.4.1 software (Martin et al. 2015) to avoid biases in the phylogenetic estimation (Schierup and Hein 2000; Arenas and Posada 2010). Finally, the VP2 dataset was comprised of 73 sequences, while the VP1 dataset considered 56 sequences, including the Chilean ones.

A maximum-likelihood tree was created using the PhyML 3.0 platform (Guindon et al. 2010) and 1,000 bootstraps were performed to support the robustness of the nodes. The tree was visualized and edited using FigTree software (Drummond and Rambaut 2007).

MEGA X was used to estimate evolutionary divergence in the VP1 and VP2 sequences (Kumar et al. 2018). The data were expressed as the relationship between the two groups located in both headers of the table, regarding average differences by group in the amino acids per each site. Standard error estimates were obtained through a bootstrapping procedure (500 replicates) and analyses were conducted using the Poisson correction model. The rate of variation between sites was modeled with a gamma distribution (shape parameter = 4).

## Results

### Samples

Fourteen viral isolates were successfully obtained from Bursas of Fabricius (BFs) from chickens, then Segments VP1 and VP2 were sequenced. Most of the examined birds’ BFs exhibited mild inflammatory lesions compatible with Gumboro disease, though their flocks of origin experienced neither mortalities nor clinical signs. This type of subclinical presentation has been previously described (Fan et al. 2019). The BF’s obtained from birds most recently showed higher inflammatory in this organ.

### Phylogenetic analysis

**Fig. 1:**
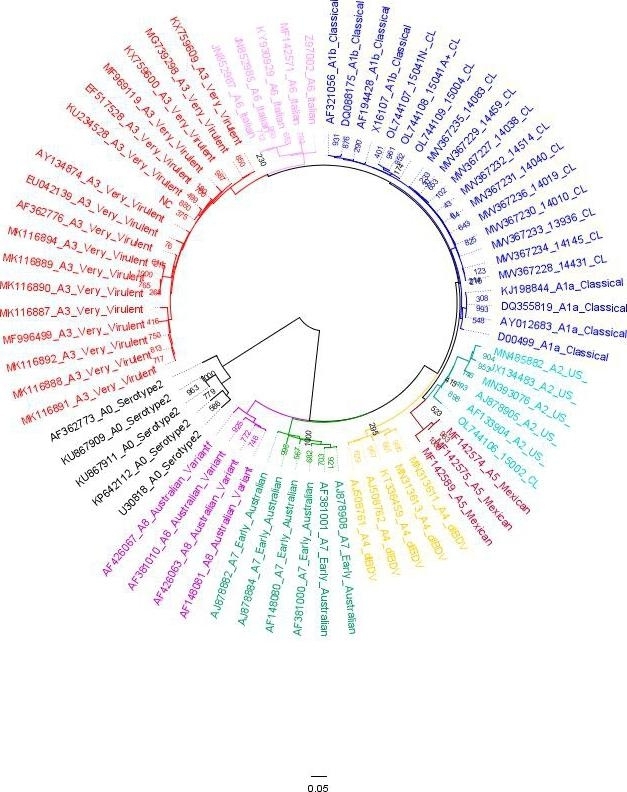
Maximum likelihood tree with 1,000 bootstrap replicates. The tree was constructed with 73 VP2 sequences, including those isolated in this study. The Chilean sequences and the genogroups 1 are in blue. The genogroups were highlighted as follows: A0 = black, A1 = blue, A2 = light blue, A3 = red, A4 = yellow, A5 = dark red, A6 = light purple, A7 = green, A8 = dark purple

Figure 1 shows thirteen Chilean IBDV sequences clustered together in an independent, well-defined clade within genogroup A1. Moreover, they were grouped with A1b_classical strains, which comprehends a lineage of classical attenuated IBDV virus within genogroup A1 (Jackwood et al. 2018). Therefore, according to Islam et al. (Islam et al. 2021), almost all Chilean sequences must be classified as A1 regarding their A segment. The A1 group contains strains previously described as classical (Michel and Jackwood 2017), though there were no clear clinical signs associated. One Chilean virus was clustered together with A2_antigenic variants strains, the sample 15002_CL. A2 group comprised antigenic variant viruses isolated mainly from the USA.

Table 2 illustrates the amino acid distance between the different genogroups based on segment A classification, Chilean sequences are an independent group, except for 15002_CL sample. “CL” column indicates the average number of per-site amino acid substitutions regarding the already described genogroups. It shows a closer relationship between Chilean sequences and A1 genogroup (0.0385) than with the remaining genogroups (A2 to A8 average = 0.0979). As it was expected, A0 genogroup was the most distant (0,2996).

**Table 2:** Amino acid distance between Chilean IBDV and the other IBDV genogroups based on segment A

	*CL*	*A0*	*A1*	*A2*	*A3*	*A4*	*A5*	*A6*	*A7*	*A8*
**CL**		0,0356	0,0103	0,0220	0,0195	0,0197	0,0235	0,0222	0,0214	0,0276
**A0**	0,2996		0,0344	0,0369	0,0355	0,0363	0,0371	0,0361	0,0368	0,0354
**A1**	0,0385	0,2976		0,0196	0,0167	0,0186	0,0221	0,0204	0,0197	0,0261
**A2**	0,0979	0,3222	0,0945		0,0223	0,0232	0,0221	0,0237	0,0251	0,0280
**A3**	0,0736	0,3130	0,0677	0,0977		0,0231	0,0260	0,0198	0,0223	0,0250
**A4**	0,0780	0,3120	0,0778	0,1073	0,0958		0,0249	0,0240	0,0227	0,0276
**A5**	0,0966	0,3255	0,0957	0,0814	0,1103	0,1076		0,0251	0,0273	0,0296
**A6**	0,0881	0,3037	0,0891	0,1123	0,0808	0,1062	0,1076		0,0241	0,0262
**A7**	0,0910	0,3149	0,0904	0,1267	0,0969	0,0945	0,1345	0,1034		0,0252
**A8**	0,1361	0,2762	0,1351	0,1462	0,1145	0,1307	0,1534	0,1197	0,1187

The number of per-site amino acid substitutions based on the average of all inter-group sequence pairs. Standard error estimates are shown above the diagonal. “CL” indicates the group of Chilean viruses.

**Fig. 2:**
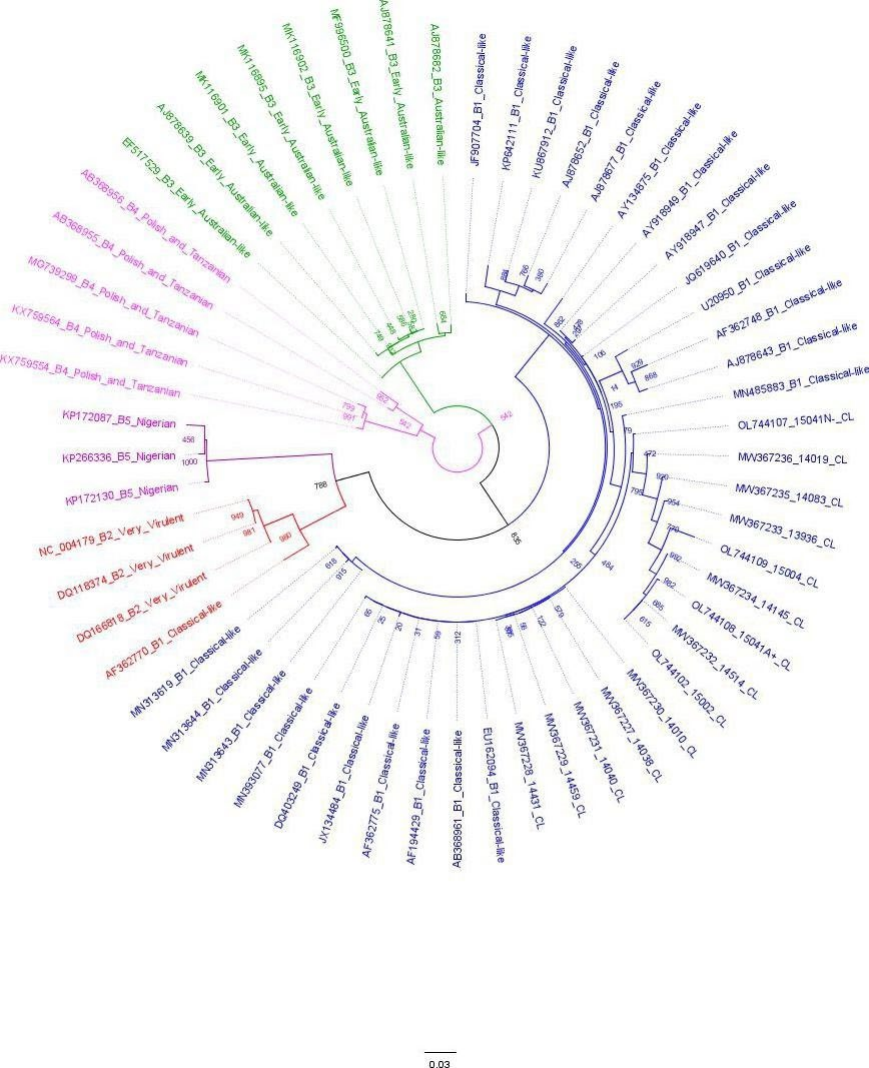
Maximum likelihood tree with 1,000 bootstrap replicates. The tree was constructed with 56 VP1 sequences, including those isolated in this study. The Chilean sequences and the genogroups 1 are in blue. The genogroups were highlighted as follows: B1 = blue, B2 = red, B3 = green, B4 = light purple, B5 = dark purple

Meanwhile, Table 3 shows the amino acidic distance of different genogroups based on segment B regarding viruses isolated in Chile. If it is true than Chilean viruses showed to be closer to the B1 genogroup, there is a minor divergence from other genogroups, although, the average number of amino acid differences per site within each genogroup is lower considering the B segment (0.0104) than the A segment (0.244). Figure 2 exhibits IBDV classification considering segment B of the genome. All Chilean Viruses were classified with B1 genogroup, which is recognized as classical strains, including the sample 15002_CL.

**Table 3:** Amino acid distance between Chilean IBDV and the other IBDV genogroups based on segment B

	*CL*	*B1*	*B2*	*B3*	*B4*	*B5*
**CL**		0,0051	0,0096	0,0114	0,0089	0,0125
**B1**	0,0164		0,0085	0,0102	0,0086	0,0122
**B2**	0,0207	0,0222		0,0116	0,0099	0,0136
**B3**	0,0306	0,0289	0,0277		0,0124	0,0149
**B4**	0,0247	0,0261	0,0267	0,0366		0,0109
**B5**	0,0344	0,0353	0,0370	0,0469	0,0318	

The number of per-site amino acid substitutions is based on the average of all inter-group sequence pairs. Standard error estimates are shown above the diagonal. “CL” indicates the group of Chilean viruses.

### Analysis of hypervariable regions

**Fig. 3:**
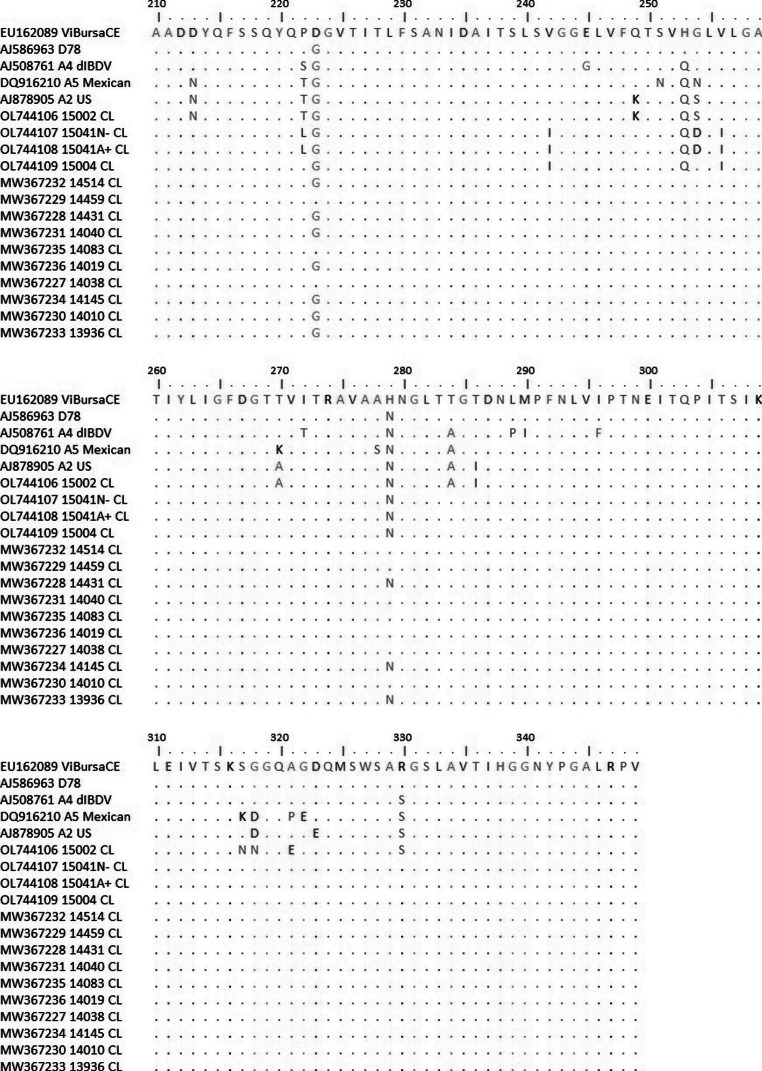
Amino acid comparison between Chilean viruses and two vaccines currently used in Chile. Dots indicate that the sample has the same amino acid as the query sequence

The comparison was conducted in the area between residues 210 and 349, a range that includes most of the hypervariable region of VP2 (Fig. 3). The Chilean viruses were compared with the D78 live attenuated vaccine (accession number AJ586963) as well as the ViBursa CE (accession number EU162089) live attenuated vaccine, both currently used in Chile. Also, were included a representative sequence from the A2 genogroup (where belongs 15002_Cl virus), a representative one from A4, and another from A5 genogroups, which have been described in America (Accession numbers AJ878905, AJ508761 and DQ916210, respectively).

Viruses 14083_CL, 14038_CL, and 14459_CL showed exactly the same sequences as the ViBursaCE vaccine. Meanwhile, viruses 14431_CL, 13936_CL, and 14145_CL showed the same sequences as the D78 vaccine. Apparently, these viruses could be vaccines re-isolated from these birds.

Notably, viruses 14010_CL, 14040_CL, 14514_CL, and 14019_CL all had a G223 like the D78 vaccine and an H279 like the VibursaCE vaccine. Position 223 has been described as relevant to determining antigenic properties in VP2 (Eterradossi et al. 1998). Position 279 has been shown to be involved in the ability to replicate in chicken embryonic fibroblasts (Lim et al. 1999). However, this does not necessarily imply a lack of pathogenicity in chickens (Abdeljelil et al. 2014). In addition, 279 residue have been described as one of the positions affected by passages in DT40 cells, which are a tumor cell line derived from the bursa of Fabricius of a chicken infected with avian leukosis virus (Terasaki et al. 2008; Delgui et al. 2009).

The viruses recently isolated in 2021 showed the same aminoacids in positions 223 y 279 as vaccine D78, although several differences were detected in residues 222, 242, 253, and 254 with D78 and Vibursa CE vaccines. Moreover, the virus 15002_CL classified as A2B1 shared the same residues as A2 representative in positions 213, 222, 249, 254, 270, 286; in addition the 330 residue is also shared with representatives from A4, which have been isolated from Brazil, Argentina, Uruguay; and A5 belonging to Mexico. These residues have been shown to be involved in antigenic variation (Fan et al. 2019).

It has been identified that residues 253 H and 284T explained mortalities of 0% (Qi et al. 2009), this combination is shown in all Chilean sequences before 2021 and the vaccines. In addition, only representatives of A2, A4, A5, and virus 15002_CL, showed an Alanine in residue 284.

Changes in residue 321 are uncommon; they would be associated with an atypical antigenic profile (Samy et al. 2020). Actually, almost all viruses showed an Alanine in this position, only the representative of A5 genogroup and the virus 15002_CL showed a Proline and Glutamic Acid in this position.

## Discussion

Apparently, Chile does not share the same viral types as other countries in the region. In Brazil, in addition to A1, genogroups 3 and 4 have been described (de Fraga et al. 2019). Meanwhile, genogroup 4 has been reported in Argentina and Uruguay (Tomás et al. 2020).

The mild lesions observed in the BF of some pullets and broilers could be from the vaccine virus-replication itself, or mild damage produced by wild viruses derived from vaccines through antigenic drift. Previously, Escaffre et al. (Escaffre et al. 2013) discussed the pathogenicity regarding both IBDV segments, and more recently Islam et al. (Islam et al. 2021) have proposed a new nomenclature considering both viral segments, understanding their contribution to viral evolution. According to these previous statements, the sample 15002_CL was classified in the genotype A2B1, pointing out a reassortment between viral segments with different origins.

Some viruses were isolated from pullets from future laying-hen flocks. Since these flocks of different ages are together in the same area, inter-flock lateral movement of viruses or vaccines is very possible. Thus, the genome recombination could be facilitated, like the 15002_CL virus, which showed a reassortment in its genome. The remaining sequences belong to viruses retrieved from broiler flocks, where strict all in–all out procedures for these chickens make lateral transmission more complicated; however, remaining or residual IBDVs from chicken flocks previously in the same barn is a real possibility. Even though the disease is not considered a serious threat in Chile, most broiler flocks are vaccinated against IBD.

Seemingly, viruses 14010_CL, 14040_CL, 14514_CL, and 14019_CL could be the result of immune escape through mutation in key residues of the wild-type virus. On the other hand, vaccine strains could have experienced a reversion to virulence due to continuous bird-to-bird passage. Viruses isolated in 2021 showed several aminoacidic differences regarding vaccines currently used in Chile, some of them are relevant in viral characteristics. For example, residue 222 has shown to be involved in viral antigenicity, its mutations had allowed viral escape from monoclonal antibodies (Eterradossi et al. 1998). In a study published by Zierenberg et al. (Zierenberg et al. 2000), the authors highlight that mutations in residues 253 and 330 were present in very virulent strains isolated from Germany and Africa, even more, Jackwood et al. (Jackwood et al. 2008) have proposed that just a mutation in residue 253 from Histidine (H) to Glutamine (Q) could increase the virulence in an attenuated strain, in the present study all of the virus isolated in 2021 carried out this aa change. Although residue 321 does not imply by itself the lack of pathogenicity of a virus, it has been shown that an Alanine in this position could be associated with a partial loss of it (Escaffre et al. 2013), only the virus 15002_CL had not an Alanine in this residue.

This is the first genetic approach to understanding the IBDV circulating in Chile. Based on the field reports and the sequences shown herein, several mutations and a reassortment process could be identified. Phylogenetic analysis suggests that the sequences retrieved in this study belong to the A1B1 and A2B1 genotypes. Estimated evolutionary divergence estimated by the amino acid distance supports this idea. While is true that VP2 sequences are a valuable phylogenetic marker, and define the recognition and binding to host receptors, RdRp encoded in the VP1 segment could change the virulence of the virus. Therefore, is recommended to use the classification given by Islam (Islam et al. 2021) instead of the previously defined by Michael and Jackwood (Michel and Jackwood 2017).

The findings made in genetic sequences are concordant with those observed in clinical samples, where Bursas of Fabricius (BF) from chickens and pullets obtained in 2017, 2018, and 2019 showed just mild lesions. On the other hand, that BF sampled in 2021 showed higher inflammatory lesions, more related to classical lesions of Gumboro Disease, nonetheless, one of them has a VP2 segment from antigenic variants. Although the viruses isolated in 2021 showed differences in several residues in comparison to vaccines (even more virus 15002_CL), it is not possible to determine precisely the protection granted by them, because it has been shown that heterologous vaccines could bring partial protection (Samy et al. 2020). However, further studies regarding the pathology of these viruses must be carried out, for a more comprehensive understanding of the genetic changes shown in this study.

Moreover, given the regional reality of neighboring countries, a surveillance program must be implemented to keep vaccination schedules up-to-date and limit concerns regarding potential clinical outbreaks of IBD, especially, with risks associated to live attenuated vaccines usage, i.e. immune escape, recombination, and reversion of virulence.

## Data Availability

The datasets generated and/or analysed during the current study are available in the National Center for Biotechnology Information repository at https://www.ncbi.nlm.nih.gov/. The accession numbers are listed in Table 1.
